# Fungal dysbiosis following antibacterial monotherapy in canine otitis externa

**DOI:** 10.1111/jsap.13801

**Published:** 2024-11-05

**Authors:** J. Juhola, E. Brennan, E. A. Ferguson, A. Loeffler, A. Hendricks, S. M. Frosini, Y. M. Chang, R. Bond

**Affiliations:** ^1^ Department of Clinical Sciences and Services University of London Hatfield UK; ^2^ Department of Pathobiology and Population Sciences University of London Hatfield UK; ^3^ Department of Comparative Biomedical Sciences, Royal Veterinary College University of London Hatfield UK

## Abstract

**Objectives:**

To evaluate evidence of *Malassezia* overgrowth following successful topical antibacterial monotherapy of refractory canine bacterial otitis using semi‐quantitative cultures.

**Materials and Methods:**

Twenty‐nine dogs with bacterial otitis were treated topically with either fluoro‐quinolone [0.5% enrofloxacin (18 dogs, 19 treatment events, 25 ears) or 0.1% marbofloxacin (1 ear), with 0.1% dexamethasone] (“FQ”) SID, or 143 mg/mL piperacillin/18 mg/mL tazobactam (“PT”) BID (11 dogs, 14 treatment events, 19 ears) for 8 to 36 days (mean 20 days). At visits 1 (V1) and 2 (V2), ear swab tips were washed in PBS + Triton X‐100 and serial dilutions spread‐plated onto blood, MacConkey (37°C, 48 hours) and modified Dixon's agar (32°C, 14 days) to generate semiquantitative counts. Microbes were identified by phenotype and MALDI‐TOF.

**Results:**

Prior to treatment, *Pseudomonas aeruginosa* was isolated alone or in combination with other bacteria in 14 FQ‐treated ears and 18 PT‐treated ears; the next most frequent bacteria were *Streptococcus canis* (8 FQ, 2 PT) and *Staphylococcus pseudintermedius* (8 FQ, 1 PT). The proportions of dogs' ears (excluding cross‐over treatments and contra‐lateral affected ears) from which bacteria were isolated were significantly reduced by treatment with both FQ (V1, 13/15; V2, 5/15) and PT (V1, 14/14; V2, 2/14). The proportions of dogs' ears from which yeasts (*Malassezia pachydermatis*, *Candida* spp.) were isolated were significantly increased by treatment in dogs treated with PT (V1, 1/14; V2, 14/14) but not FQ (V1, 3/15; V2, 6/15).

**Clinical Significance:**

Otitis cases that receive potent antibacterial monotherapy must be monitored for yeast overgrowth.

## INTRODUCTION

The surface of healthy mammalian skin supports a diverse array of resident microbes and parasites that form a dynamic, interactive ecosystem that, under normal circumstances, is homeostatically restricted by host defences mediated by various physical, chemical and immunological processes (Jenkinson, 1992; Mason et al., 1996; Saijonmaa‐Koulumies & Lloyd, 1995). Resident microbes such as bacteria and yeasts tend to exist as mixed microcolonies that manifest crosstalk and cross‐regulation by exchange of metabolites with both synergistic and inhibitory effects, maintaining a balanced population (Allaker et al., 1988; Allaker & Noble, 1992; Saijonmaa‐Koulumies & Lloyd, 1995). Normal microbial colonisation by resident organisms protects the host from potentially pathogenic visiting microbes, but residents also form a reservoir of potential pathogens (such as staphylococci and *Malassezia*) that may opportunistically cause disease (Jenkinson, 1992; Mason et al., 1996; Saijonmaa‐Koulumies & Lloyd, 2002). Recent studies involving sequencing and metagenomics have revealed more diverse cutaneous microbial populations than those defined by traditional cultural methods (Leonard et al., 2022; Ngo et al., 2018; Puigdemont et al., 2021; Secker et al., 2023). In mammalian microbial ecology, the term “dysbiosis” is most often used in the context of diseases associated with an imbalance in the intestinal microbiota (El Mouzan et al., 2018; Liguori et al., 2016; Sokol et al., 2017), but it is equally applicable to the skin and ear, where studies in atopic dermatitis predominate (Meason‐Smith et al., 2015; Meason‐Smith et al., 2019; Ngo et al., 2018; Rodrigues Hoffmann, 2017; Secker et al., 2023).

We have observed, by routine cytological examinations, that *Malassezia* yeast overgrowth (ie a fungal dysbiosis) is not an infrequent sequel to successful topical therapy of refractory canine bacterial otitis externa by use of potent antibacterial drugs, such as enrofloxacin or piperacillin‐tazobactam. We often use these products after the conventional licensed polypharmacy ear products have been ineffective and/or when their use is contraindicated due to suspected or observed absence of an intact tympanic membrane, in accordance with responsible antimicrobial stewardship. There are sporadic reports of an association between the use of fluroquinolone drops (ofloxacin or ciprofloxacin) and the development of otomycoses in human otitis (Jackman et al., 2005; Martin et al., 2005; Schrader & Isaacson, 2003). Since this phenomenon is not well described in the veterinary literature, we hypothesised that these observations would be confirmed prospectively in clinical cases of canine otitis externa by cultures of ear swabs using a semi‐quantitative swab wash method.

## MATERIALS AND METHODS

### Ethics and subject dogs

The study was approved by the Royal Veterinary College’s Clinical Research Ethical Review Board (URN2020‐1953‐2). Dogs whose ears were treated topically with compounded fluoroquinolone (FQ) solutions or piperacillin‐tazobactam were prospectively recruited following signed owner consent. From a review of previous case data, we anticipated 20 to 25 eligible cases per annum with a frequency of yeast overgrowth of 67% after antibiotic monotherapy (observed in 20 out of 30 previously treated ears). Based on this frequency and an expected 50 ears requiring topical treatment with antibiotic monotherapy [topical therapy is preferred to systemic therapy whenever possible (Nuttall, 2016)], the minimum required sample size for estimating the rate of yeast overgrowth as an adverse event with 95% confidence level and 15% absolute precision was 22 ears (Dhand & Khatkar, n.d.). Allowing for study attrition, the goal was to recruit all consecutive cases that were presented to our clinic and met the inclusion criteria between January 2022 and September 2023. Inclusion criteria comprised a dog with chronic (>4 weeks) unilateral or bilateral otitis externa and/or middle ear disease, cytological and/or culture‐based evidence of an otic bacterial infection (with zero or only rare yeasts observed cytologically), a poor response to prior conventional topical polypharmacy ear treatments and/or a contra‐indication for their use (primarily suspected or observed absence or rupture of the tympanic membrane), with an owner prepared to administer the (unlicensed) topical formulation. Exclusion criteria comprised the inability to obtain and process the necessary swab samples from the patient, and the need for any adjunctive topical therapy in the opinion of the attending clinician. Risk of ototoxicity should these products access the middle ear was deemed very low based on previous safety studies with FQ/dexamethasone and piperacillin‐tazobactam in laboratory animals (Daniel & Munguia, 2008; Gates, 2001; Jang et al., 2008; Lemke et al., 2009), and our own and others' clinical experience in dogs (Gotthelf, 2004; Nuttall, 2016; Paterson, 2018).

### Topical antibacterial products

**FIG 1:**
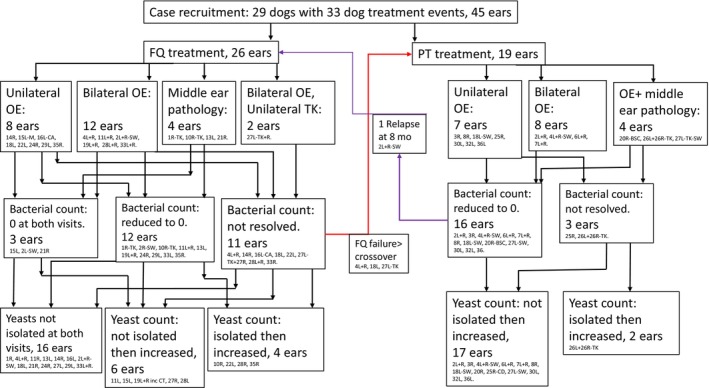
Flowchart illustrating the treatment allocations, ear pathology (otitis externa and or middle ear disease) and the effects of antibacterial treatment (fluroquinolone, FQ; piperacillin‐tazobactam, PT) on bacterial and yeast colony counts in 29 dogs (33 treatment events, 45 ears). BSC Basosquamous carcinoma, CA Ceruminous adenoma, L Left, M Marbofloxacin, R Right, SW Cross‐over from other treatment group, TK Tympanokeratoma.

The dogs' ears were treated topically with either a FQ solution (0.5% to 1.0% enrofloxacin or 0.1% marbofloxacin, with 0.1% dexamethasone sodium phosphate) once daily (0.5 to 1 mL), or 143 mg/mL piperacillin/18 mg/mL tazobactam (PT) twice daily (Fig 1). The enrofloxacin solution was prepared by diluting 2 to 4 mL of injectable enrofloxacin (Baytril 25 mg/mL Solution for Injection; Elanco UK AH Ltd) and 5 mL of 0.2% dexamethasone sodium phosphate (Colvasone 0.2% w/v Solution for Injection; Norbrook Laboratories Ltd) in 1 to 3 mL of sterile water for injection. The marbofloxacin solution was prepared by diluting 1 mL of a 10 mg/mL solution of marbofloxacin (Marbocyl™ SA 200 mg Powder and Solvent for Solution for Injection; Vetoquinol UK Ltd.) and 5 mL of 0.2% dexamethasone sodium phosphate (Colvasone 0.2% w/v Solution for Injection; Norbrook Laboratories Ltd) in 4 mL sterile water for injection. The piperacillin/tazobactam solution was prepared by reconstituting a vial containing 2 g of piperacillin and 0.25 g of tazobactam (Piperacillin 2 g/Tazobactam 250 mg powder for solution for infusion vials; Bowmed Ibisqus Ltd) in 14 mL of sterile water for injection; each reconstituted vial was kept in a refrigerator and the contents used (0.5 to 1 mL twice daily) or discarded within 5 days.

### Treatment allocation and assessments

The initial choice of drug was not randomised and depended on the preference of the attending clinician, although PT was reserved for the cases deemed to be most severe or refractory to other treatments. The aim was to repeat the clinical examination and ear sampling after 2 to 3 weeks of treatment, although the timing of the re‐examination depended in part upon dog owner availability. Whilst clinical and cytological assessments occurred at each visit (Table 1), the primary measure of antibacterial efficacy was by semi‐quantitative bacterial culture. A reduction of the bacterial culture count to zero was considered a treatment success, whereas failure was represented by continued isolation of bacteria despite the treatment.

**Table 1:** Association between the isolation of bacteria or yeast and their observation in cytological specimens from the ear canals (*n* = 45) of 29 dogs with chronic otitis before [visit 1 (V1)] and after [visit 2 (V2)] topical treatment with fluoroquinolones (FQ) or piperacillin/tazobactam (PT)

Observations	FQ‐bacteria		FQ‐yeast		PT‐bacteria		PT‐yeast	
	V1	V2	V1	V2	V1	V2	V1	V2
Isolated and observed	20	8	0	5	19	0	0	16†
Not isolated, not observed	2	15	21	16	0	14	16	0
Isolated and not observed	3	3	4	5	0	3	2	3
Not isolated but observed	1	0	1	0	0	2‡	1	0
Total	26	26	26	26	19	19	19	19
Percentage agreement§	84.6	88.5	80.8	80.8	100	73.7	84.2	84.2

^†^ Includes one case where budding yeasts of a morphology distinct from *Malassezia* sp. were observed (*Candida dubliniensis* isolated)

^‡^ Intervals from last treatment to swab sampling for culture were 4 and 24 hours

^§^ Calculated by the formula: Percentage agreement = (microbe isolated and observed + microbe not isolated and not observed) × 100/total

Antimicrobial susceptibility testing was performed on bacterial isolates but therapeutic decisions were not based on these data since breakpoints for topical therapy are not available; results reported by laboratories for systemic therapy cannot be interpreted in a meaningful way for topical drug use. The proper application of veterinary‐specific breakpoints requires drug concentrations at the site of infection to reach or exceed expected values following systemic administration to animals with normal renal function (Clinical and Laboratory Standards Institute, 2023). Accordingly, and since the response to therapy in otitis cases is best assessed using clinical criteria and cytology (Nuttall, 2016), these susceptibility data are not reported here. Since recent antibacterial therapy might interfere with subsequent bacterial cultures, owners were invited to stop treatment 24 hours before the follow‐up visits.

### Concurrent treatments

Thirteen of the 19 (68%) FQ‐treated dogs and 12 of the 14 (86%) PT‐treated dogs received glucocorticoids orally to address ear canal hyperplasia/stenosis and or to manage a hypersensitivity disorder as a primary factor in their otitis externa. During treatment with either FQ or PT, 24 dogs received oral prednisolone either once daily or on alternate days (where given, mean dose = 1.2 mg/kg/48 hours, range 0.3 to 2.4 mg/kg/48 hours). One dog which did not tolerate prednisolone (lethargy, marked polyuria/polydipsia) received oral dexamethasone at 0.06 mg/kg every other day. One FQ‐treated dog (D28) received concurrent oral cefalexin for superficial pyoderma and levothyroxine for hypothyroidism but none of the remaining study dogs received either oral antibacterial or antifungal drugs.

Most dogs underwent general anaesthesia for video‐otoscopy and ear flushing (n = 23) combined with computed tomography of the tympanic bullae (n = 20). The ear flushing event immediately preceded the introduction of the FQ or PT therapy on 11 occasions; flushing occurred 1 to 6 weeks (mean 3 weeks) after starting FQ or PT treatment on 11 occasions (reflecting need for antibacterial treatment in the interval from first examination to re‐visit for planned ear procedure). On four occasions, flushing preceded FQ or PT treatment by 1 to 9 weeks (mean 4.0 weeks)[reflecting introduction of either FQ or PT after post‐flush treatment failure with alternative products]. Other than either FQ or PT, no other ear treatments were administered.

### Cytological sampling and semi‐quantitative cultures

At consecutive visits before and after treatment, an ear swab was collected from each affected ear for cytological evaluation and for semi‐quantitative culture. Swabs were rolled on a glass slide and stained with a modified Wright's stain for assessment of number and type of microbes. Slides were examined using a light microscope with a ×100 oil immersion objective; populations of each type of microbe (coccus, rod, *Malassezia* or other yeast) were subjectively graded on a scale of 0, +, ++, +++, ++++ (Budach & Mueller, 2012).

Cultures were obtained from affected ears by the application of a viscose‐tipped flexible polypropylene swab (TS/17‐A250, Technical Service Consultants Ltd, Heywood, Lancs, UK) to the external ear canal for 5 seconds (Allaker et al. 1992, Bond et al. 1996, Bond et al. 1995). The culture swab tips were washed in 2 mL of a “wash fluid” comprising 0.075 M phosphate‐buffered saline +0.1% Triton X‐100 (Williamson & Kligman, 1965) for 30 seconds and 25 μL aliquots of serial 10‐fold dilutions prepared in “diluting fluid” (0.037 M phosphate‐buffered saline +0.05% Triton X‐100) (Williamson & Kligman, 1965) were spread‐plated in duplicate onto quadrants of 90 mm Petri dishes containing blood agar, MacConkey agar (37°C for 48 hours) and modified Dixon's agar (Gueho‐Kellerman et al., 2010) (32°C for 14 days). The bacterial or yeast count obtained by each swab was calculated by multiplying the mean colony count from each paired and duplicate quadrant of agar by a constant of 80 [25 μL (1/80th) of the 2 mL wash fluid sample plated] and by the lowest dilution factor that allowed individual colonies to be clearly identified and counted. Counts were expressed as the log_10_(colony‐forming units [CFU]/swab + 1). Since viable microbial counts on swab tips might show either reduced viability or proliferation, the goal was to process the swabs as soon as possible after sampling, with a target time of less than 5 hours. Microbes were identified by phenotype and matrix‐assisted laser desorption/ionisation‐time of flight (MALDI‐TOF) (Vitek MS; bioMérieux UK Ltd).

### Statistical analyses

**FIG 2:**
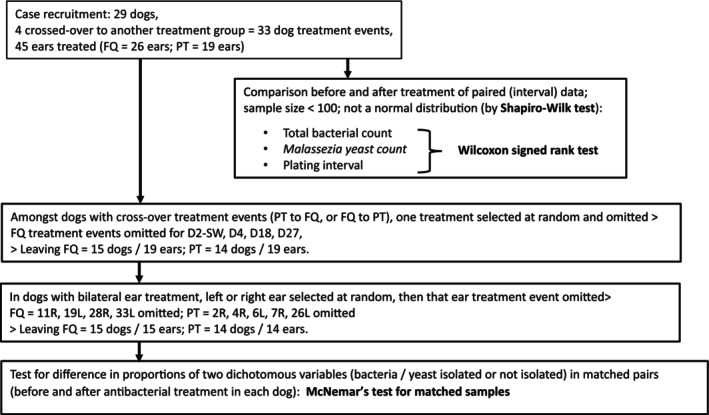
Flowchart illustrating the methods used in the statistical analysis of the results of a study of the effects of antibacterial treatment (fluroquinolone, FQ; piperacillin‐tazobactam, PT) on bacterial and yeast counts in 29 dogs (33 treatment events, 45 ears) with chronic otitis. L Left, R Right, SW Cross‐over from other treatment group.

Population sizes of bacteria and *Malassezia* in each ear, and swab sample plating intervals for each dog, were compared before and after treatment using Wilcoxon signed rank tests; the Shapiro Wilk test was used to determine that these data were not normally distributed (Fig 2). McNemar's tests for matched samples were used to compare the proportions of dogs from which bacteria or yeasts were either isolated or not isolated before (visit 1) and after (visit 2) the administration of the antibacterial monotherapy (Bland, 1987). In order to avoid confounding factors from the cross‐over between treatment groups that occurred in four cases, one treatment event only (either FQ or PT, not both) was selected at random (by toss of a coin) for analysis of these dichotomous data in each of these four dogs (Fig 2). Similarly, to address the potential for confounding effects from cases with bilateral rather than unilateral otitis treatment, data from either the left or the right ear only were selected at random, also by toss of a coin (Fig 2). Analyses were performed using the Unistat v3 statistical software package (Unistat Ltd., London), with P < 0.05 for significance. The percentage agreements between the presence of bacteria by culture and cytology, and yeast by culture and cytology, before and after treatment were calculated according to the methods of McHugh (McHugh, 2012; Table 1).

## RESULTS

### Subject dogs, treatment allocation and assessments

Twenty‐nine dogs were enrolled, comprising 20 breeds or crossbreeds; breeds represented by more than one case included Cocker spaniels (*n* = 7), Labrador retrievers (*n* = 2) and basset hounds (*n* = 2). There were four entire males, 14 neutered males, one entire female and 10 neutered females, aged between 1 year 8 months and 11 years 7 months (median 6 years 9 months). Duration of ear disease ranged from 2 to 84 months (median 10 months). The dogs primarily presented with unilateral or bilateral otitis externa although three dogs had concurrent otitis externa and middle ear disease, and four had middle ear disease only (Fig 1). Of the seven dogs with middle ear disease, four had tympanokeratoma (cholesteatoma), one had a basosquamous carcinoma expanding the middle ear and another had a history of removal of two grass seeds from the external canal; three of these middle ear cases presented with concurrent vestibular signs. All but two of these dogs had received prior topical otic antifungal therapy (miconazole, clotrimazole, nystatin or terbinafine).

There were 33 dog treatment events involving 45 ears; 19 dogs (26 ears) received FQ treatment (25 enrofloxacin and one marbofloxacin) and 14 dogs (19 ears) received PT (Fig 1). The FQ treatment duration ranged from 8 to 32 days (mean ± SD = 20.5 ± 7.2). The PT treatment duration ranged from 10 to 36 days (mean ± SD = 20.2 ± 6.2).

The study population included one dog (D2) that was first treated successfully with PT and then treated with FQ 8 months later following an episode of otitis externa associated with a different bacterial pathogen, and three dogs that were immediately switched to PT following FQ treatment failure (D4, D18 and D27) (Fig 1). Five dogs (two PT and three FQ) that presented during the study period were not included as the attending clinician dispensed adjunctive topical otic treatments and one further dog (FQ) was omitted as samples could not be obtained and processed.

### Bacterial populations before and after treatment

The interval from sampling to plating the swab‐wash dilutions ranged from 25 to 240 minutes. The median (lower‐upper quartile) intervals at the first (90 minutes; 65 to 133) and second visits (85 minutes; 60 to 120) did not vary significantly (P = 0.56, Wilcoxon signed rank test). Amongst the 33 dog treatment events, the interval from last topical ear treatment to sampling at the first visit in 19 treated dogs (data not recorded in one dog) ranged from 3 to 96 hours (median 24 hours; lower‐upper quartile: 20 to 48 hours); 13 dogs had not received topical ear treatment in the 7 days prior to first sampling. The interval from last topical ear treatment to sampling at the second visit (data not recorded in four dogs) ranged from 2 to 168 hours (median 24 hours; lower‐upper quartile: 12 to 24 hours).

**FIG 3:**
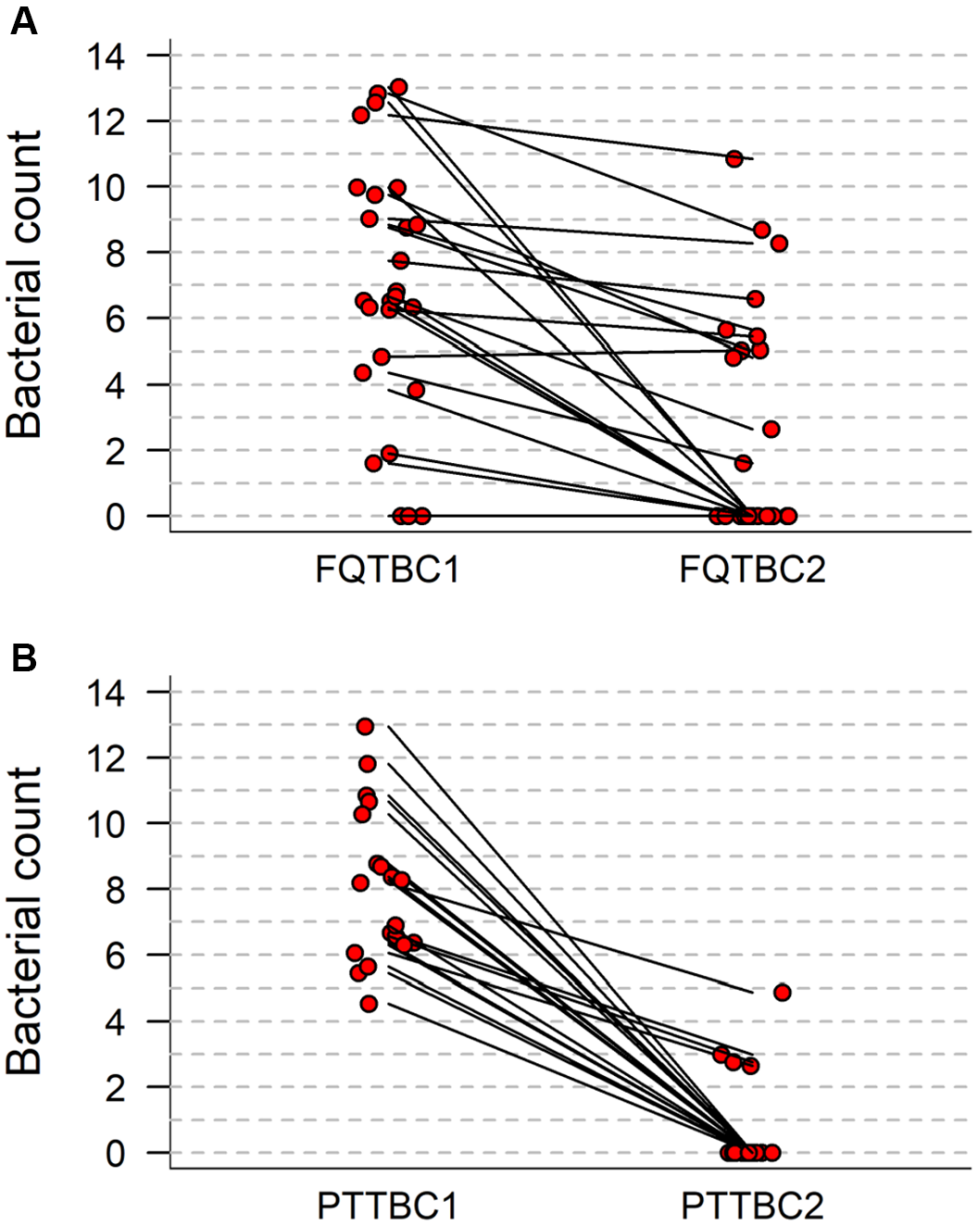
(A) Total bacterial counts (log_10_[CFU/swab+1]) before and after topical fluoroquinolone therapy in 19 dogs (26 ears) with otitis externa/middle ear disease. (B) Total bacterial counts (log_10_[CFU/swab+1]) before and after topical therapy with piperacillin/tazobactam in 14 dogs (19 ears) with otitis externa/middle ear disease.

Prior to treatment, *Pseudomonas aeruginosa* was isolated alone or in combination with other bacteria in 14 out of 26 FQ‐treated ears and 18 out of 19 PT‐treated ears; the next most frequent bacteria were *Staphylococcus pseudintermedius* (eight FQ‐treated ears and one PT‐treated ear) and *Streptococcus canis* (eight FQ‐treated ears and two PT‐treated ears; Table 2). Amongst all 45 treated ears, pre‐treatment total bacterial counts, expressed as log_10_[CFU/swab+1], were typically high with a median count (lower, upper quartile) of 6.81 (6.06, 9.75) and with a range of 0 to 13.04 (Fig 3). Bacteria were not isolated in swab samples from three external ears at visit 1 (Figs 1 and 3A); these cases included two cases of middle ear disease with bacteria recovered from deeper samples.

**Table 2:** Bacteria isolated using a semi‐quantitative swab wash method prior to topical treatment with either enrofloxacin or marbofloxacin (FQ) or piperacillin/tazobactam (PT) for canine otitis externa in 45 ears

Bacteria isolated	Number treated with FQ	Number treated with PT
*Pseudomonas aeruginosa* (PA)	3	14
PA + *Streptococcus canis* (SC)	6	2
PA + SC + *Pluribacter gergoviae*	2	0
PA + *Staphylococcus pseudintermedius* (SP)	2	1
PA + SP + *Corynebacterium auriscanis* (CA)	1	0
PA + *Enterococcus faecalis* (EF)	0	1
SP	2	0
SP + *Micrococcus luteus*	1	0
SP + CA + *Enterococcus faecalis*	1	
SP + EF+ *Proteus mirabilis*	1	0
CA + *Staphylococcus schleiferi* (SS)	0	1
CA + SS + *Proteus mirabilis*	1	0
*Proteus mirabilis*	1	0
*Staphylococcus epidermidis*	1	0
*Staphylococcus epidermidis* + *Micrococcus luteus*	1	0
No bacteria cultured	3	0

After FQ treatment, both bacterial culture counts and cytological scores were reduced to zero in 10 dogs (12 ears) out of 19 dogs (26 ears); bacterial culture counts remained at zero in three ears (Figs 1 and 3A‐2, Tables 1 and 3). PT treatment reduced bacterial culture counts and cytology scores to zero in 10 dogs (14 ears), and bacterial culture counts to zero with low numbers (+, ++) of residual bacteria in cytological specimens from two dogs (two ears) (Figs 1 and 3B, Tables 1 and 3). In the three dogs (four ears) treated with PT after FQ treatment failure, PT treatment was successful in each ear. Initial bacterial counts (median log_10_[CFU/swab+1], lower‐upper quartile) (FQ: 6.59, 4.36 to 9.75; PT: 8.19, 6.30 to 10.28) were significantly (FQ, P < 0.0001;PT, P = 0.0001) reduced at follow‐up sampling in both treatment groups (FQ: 0, 0‐5.04; PT 0, 0‐0) (Fig 3). The overall percentage agreement between the presence of bacteria on culture and cytology (both treatment groups, before and after therapy) was 87%.

**Table 3:** Proportions of dogs from which bacteria were either isolated or not isolated before [visit 1 (V1)] and after [visit 2 (V2)] the administration of antibiotic monotherapy (FQ, fluoroquinolone; PT, piperacillin‐tazobactam) for the treatment of chronic otitis in 29 dogs

	FQ treated dogs		PT‐treated dogs		FQ + PT‐treated dogs	
	V2 bacteria isolated	V2 bacteria not isolated	V2 bacteria isolated	V2 bacteria not isolated	V2 bacteria isolated	V2 bacteria not isolated
V1 bacteria isolated	5	8	2	12	7	20
V1 bacteria not isolated	0	2	0	0	0	2
Significance (P value)*	0.008		0.0005		<0.0001	

* McNemar's test for matched pairs: the null hypothesis is that the proportions of dogs which bacteria are either isolated or not isolated before (V1) and after (V2) antibiotic monotherapy are the same

### Yeast populations before and after treatment

**FIG 4:**
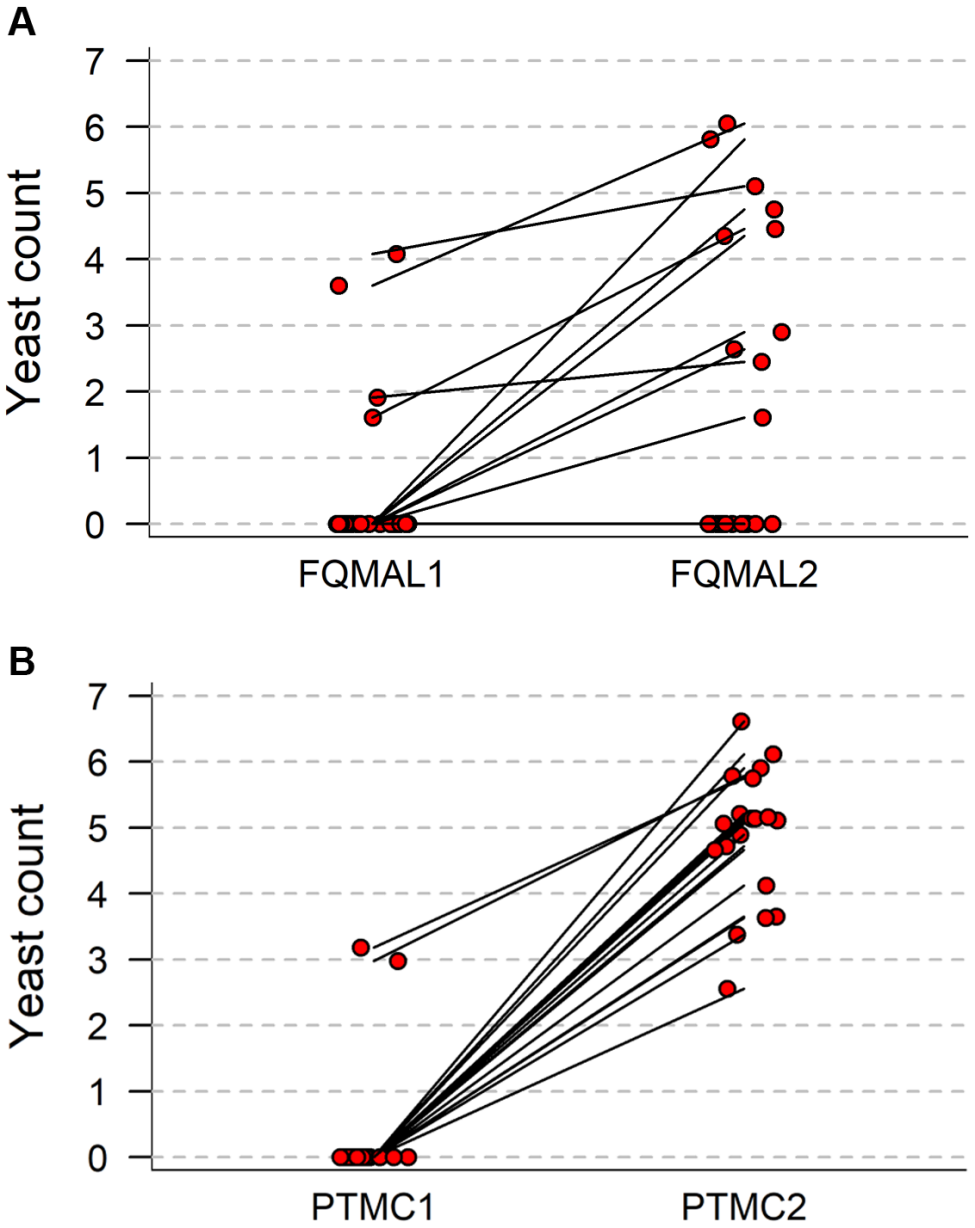
(A) Yeast (*Malassezia pachydermatis*) counts (log_10_[CFU/swab+1]) before and after topical fluoroquinolone therapy in 19 dogs (26 ears) with otitis externa/middle ear disease. (B) Yeast (*Malassezia pachydermatis*, *Candida* spp.) counts (log_10_[CFU/swab+1]) before and after topical therapy with piperacillin/tazobactam in 14 dogs (19 ears) with otitis externa/middle ear disease.

*Malassezia pachydermatis* was isolated, in counts ranging from 1.61 to 4.08 (median 3.05), prior to treatment from 4/19 FQ‐treated dogs (4/26 ears, Figs 1 and 4A) and from one of 14 PT‐treated dogs (2/19 ears, Figs 1 and 4B); yeasts were observed by pre‐treatment cytological examination in two of these cases (Table 1). After treatment, *M. pachydermatis* was isolated from eight of 19 FQ‐treated dogs (10/26 ears, Figs 1 and 4A, Table 1). Yeasts were isolated from all PT‐treated dogs after treatment; *M. pachydermatis* was isolated from 13 of 14 dogs (18/19 ears) and *Candida dubliniensis* was isolated from one ear (D25R) (Fig 4B). Antibacterial treatment was associated with an increased frequency of isolation of *M. pachydermatis* in the PT‐treated ears (Table 4) but not in FQ‐treated ears whose bacteriological response was less often satisfactory. Overall, *M. pachydermatis* counts (median, lower‐upper quartile) (FQ: 0, 0‐0; PT: 0, 0‐0) were significantly (FQ, P = 0.0051; PT, P = 0.0003) increased post‐treatment in both groups (FQ: 0, 0 to 2.90; PT 5.06, 3.63 to 5.21). In each case where *M. pachydermatis* was isolated at the first visit, the initially low populations increased following treatment with either FQ (four dogs, four ears) or PT treatment (one dogs, two ears); population increases ranged from 3.5 to 720‐fold (median × 325).

**Table 4:** Proportions of dogs from which yeasts (*Malassezia pachydermatis*, *Candida* spp.) were either isolated or not isolated before [visit 1 (V1)] and after [visit 2 (V2)] the administration of antibiotic monotherapy (FQ, fluoroquinolone; PT, piperacillin‐tazobactam) for the treatment of chronic otitis in 29 dogs

	FQ treated dogs		PT‐treated dogs		FQ + PT‐treated dogs	
	V2 yeast isolated	V2 yeast not isolated	V2 yeast isolated	V2 yeast not isolated	V2 yeast isolated	V2 yeast not isolated
V1 yeast isolated	3	0	1	0	4	0
V1 yeast not isolated	3	9	13	0	16	9
Significance (P value)*	0.25		0.0002		<0.0001	

* McNemar's test for matched pairs: the null hypothesis is that the proportions of dogs from which yeasts are either isolated or not isolated before (V1) and after (V2) antibiotic monotherapy are the same

*Candida tropicalis* was isolated from both the left (3.53) and right (4.62) ears in one dog that also developed profuse *M. pachydermatis* growth (left, 4.75; right, 5.81) after successful FQ‐treatment for bilateral infections with both *P. aeruginosa* and *S. canis*. *Candida dubliniensis* was the only yeast isolated in profuse culture (5.90) in one PT‐treated dog whose *P. aeruginosa* infection responded only partially to treatment (pre‐treatment count, 6.06; post‐treatment 2.64).

Of the six ears that yielded yeast isolates when cultured before treatment, none had cytological evidence of yeast; two ears from which *M. pachydermatis* was not isolated showed low numbers of yeasts on cytology (Table 1). Of the 29 ears that yielded yeast isolates after treatment, eight had no cytological evidence of yeast. The overall percentage agreement between the presence of yeasts on culture and cytology (both treatment groups, before and after therapy) was 82% (Table 1).

Of the 29 ears from which yeasts were isolated at the second visit, 22 were treated with a topical antifungal medicinal product because the clinician regarded the post‐antibacterial yeast overgrowth to be of clinical significance. The topical products prescribed were a clotrimazole 1% solution (Canesten, Bayer; *n* = 7), a miconazole, gentamicin and hydrocortisone aceponate‐containing product (EasOtic, Virbac, *n* = 7); a posaconazole, orbifloxacin and mometasone furoate‐containing product (Posatex, MSD Animal Health, *n* = 6), and a terbinafine, florfenicol and mometasone furoate‐ containing product (Neptra, Elanco UK AH Ltd., *n* = 2). One dog with yeast overgrowth was treated surgically because of tympanokeratoma. Six ears (five dogs) received monotherapy with topical glucocorticoid on the basis that control of otic inflammation would be sufficient to limit yeast overgrowth (Rigaut et al., 2024); four of these had no yeasts observed on cytology despite positive cultures, and one had a record of a low number of yeasts (+on cytology). All six of these ears had no yeasts observed by cytological examination at their next follow‐up visit 4 to 8 weeks later.

### Tolerability of the ear treatments

No adverse effects were noted with the FQ and PT treatments used in this study.

## DISCUSSION

Our study demonstrates that effective antibacterial monotherapy is associated with *Malassezia* (and less often *Candida*) overgrowth in dogs with bacterial otitis. We speculate that this is most likely due to the suppression of commensal and pathogenic bacteria that normally have a competitive and inhibitory effect on yeast growth, and potentially ongoing primary or perpetuating pathological processes within these chronically diseased ears. While we are unaware of previous reports of this observation in canine patients, there are sporadic reports of similar outcomes in human otitis wherein fluroquinolone drops (ofloxacin or ciprofloxacin) were associated with otomycoses (Jackman et al., 2005; Martin et al., 2005; Schrader & Isaacson, 2003). In a paediatric otolaryngology clinic, the incidence of fungal otitis externa or media, primarily associated with *Candida albicans*, *C. parapsilosis* or *Aspergillus* species, showed a statistically significant increase in the 3 years immediately following the introduction and widespread use of FQ (ofloxacin) ototopical drops (Martin et al., 2005). An increased incidence of otomycosis was similarly reported in two other paediatric clinics (Jackman et al., 2005; Schrader & Isaacson, 2003). These studies show striking similarities to our observations in dogs; topical FQ (and in our case also piperacillin‐tazobactam) therapy is often effective in both human and canine bacterial otitis, but careful post‐treatment assessment for otomycoses is warranted. Cytological assessment allows monitoring for effective clearance of both the original bacterial pathogen(s) but also for proliferation of opportunistic yeasts.

One notable difference between the human studies and the present investigation is the predominance of *Candida* and *Aspergillus* in human patients, whereas in dogs the yeast overgrowth was primarily associated with *M. pachydermatis* and only occasionally *Candida*. In the three human studies cited above, the conditions used to culture fungi were not specified; failure to recover *Malassezia* may therefore reflect use of only conventional culture media and not the selective complex media supplemented with lipids required for isolation of *Malassezia* species known to colonise human skin and ears (Alshahni et al., 2021; Gueho‐Kellerman et al., 2010). Previously, some authors have speculated that long‐term use of antibacterials might promote yeast overgrowth by altering the pH of the ear canal (Alshahni et al., 2021), although others have stated that the skin of the ear has a significant buffering capacity (Nuttall, 2016).

It is important to emphasise that the antibacterials used in this study are not appropriate for routine use in conventional canine otitis cases. Rather, the treated animals in this study were mostly referred because of chronic otitis externa with treatment failure using licensed products, or otitis media where the licensed products are contra‐indicated. Aqueous solutions of injectable enrofloxacin are often used topically in referral practice by dermatology specialists, primarily for cases of *Pseudomonas* otitis in dogs, in view of its potential activity against an array of Gram‐negative pathogens including *Pseudomonas aeruginosa* and the Enterobacterales (Nuttall, 2016), and apparent safety in the middle ear as discussed above.

Piperacillin‐tazobactam is an ureidopenicillin/beta‐lactamase inhibitor combination whose spectrum of antibacterial activity includes Gram‐positive and Gram‐negative aerobic and anaerobic bacteria (Gin et al., 2007). This drug is primarily used in human hospitals where its spectrum of activity and tolerability makes it a reliable choice in moderate‐severe infections of skin and soft tissues, and lower respiratory and urogenital systems (Gin et al., 2007). Our study indicates that this drug is very frequently effective as a “last‐line” topical antibacterial drug in cases of bacterial otitis externa and middle ear disease, including in cases where FQs have failed, and where total ear canal ablation or euthanasia would otherwise be required. Veterinary surgeons who practice within the European Union should be aware that ureidopenicillins (including piperacillin) are listed in the European Union's Annex of Commission Implementation Regulation and are therefore banned from use in any animal species in those countries (Schmerold et al., 2023). Accordingly, responsible antimicrobial stewardship indicates that PT should only be used as a drug of last resort for the secondary bacterial infection in cases of canine otitis (provided there are no national legislative impediments) and where there is also detailed evaluation and management of the concurrent predisposing, primary and perpetuating factors involved in the otitis, to maximise the potential for a positive clinical outcome.

One limitation of this study was the occasional failure of owners to follow our request that topical therapy was withdrawn 24 hours before sampling at the follow‐up visit. Whilst swab transfer of residual antibacterial agent from a treated ear to the bacterial culture plates is possible, the swab‐wash process would have a dilution effect on any inhibitory activity. Furthermore, the rare observation of bacteria in cytological specimens from ears with no bacterial isolates (only two examples out of 31 culture negative ears at visit 2, Table 1) indicates that any potential inhibitory effect was rarely, if ever, present. The primary objective of this study was the quantification of yeasts after antibacterial treatments and the inadvertent swab transfer of antibacterial drugs would not be expected to have any effect on fungal growth.

The concurrent use of topical dexamethasone in combination with the FQ antibacterials followed our usual clinical practice; the antipruritic and anti‐inflammatory effects of topical glucocorticoid therapy are beneficial in most cases of canine otitis and media (Miller et al., 2013; Rigaut et al., 2024). It is not certain whether the more frequent antibacterial efficacy of PT reflects an inherently greater activity against the pseudomonads and Gram‐positive cocci isolated from the PT‐treated dogs, or the absence of concurrent topical dexamethasone. A systematic literature search on the use of ciprofloxacin 0.3% and dexamethasone 0.1% otic suspension in human paediatric patients provided clear evidence to support the inclusion of dexamethasone in infected cases of otitis externa and media (Wall et al., 2009). In canine *Malassezia* otitis externa, a combination of glucocorticoid, azole antifungal and antibacterial had a superior clinical effect when compared with an azole alone (Bensignor & Grandemange, 2006). Our observation of an anti‐*Malassezia* effect with topical glucocorticoid alone in six ears is in accordance with a recent large randomised, controlled trial (Rigaut et al., 2024).

The allocation of the two types of antibacterial drugs was not randomised and therefore any comparisons between the two treatment groups in terms of bacteriological efficacy must be treated with caution. However, PT is regarded within our team as being a “last line” drug that tends to be selected for the most severe cases, for reasons mentioned above, and the observed bacteriological efficacy was impressive.

In conclusion, these data confirm the hypothesis that yeast populations may opportunistically increase in dogs' ears following treatment with highly efficacious broad spectrum antibacterial monotherapy. Topically applied piperacillin/tazobactam was especially efficacious in treating *Pseudomonas* otitis externa in the subject dogs. Future studies are required to determine whether antifungal drugs or antiseptic ear rinses can be combined with FQ and PT to prevent the fungal dysbiosis observed here. Otitis cases that receive potent antibacterial monotherapy must be monitored for yeast overgrowth.

### Author contributions

**J. Juhola:** Data curation (equal); formal analysis (equal); investigation (equal); resources (equal); visualization (equal); writing – original draft (equal). **E. Brennan:** Investigation (equal); resources (equal); writing – review and editing (equal). **E. A. Ferguson:** Investigation (equal); resources (equal); supervision (equal); writing – review and editing (equal). **A. Loeffler:** Conceptualization (equal); funding acquisition (equal); investigation (equal); resources (equal); supervision (equal); writing – review and editing (equal). **A. Hendricks:** Conceptualization (equal); formal analysis (equal); funding acquisition (equal); investigation (equal); resources (equal); supervision (equal); writing – original draft (equal). **S. M. Frosini:** Conceptualization (equal); funding acquisition (equal); investigation (equal); writing – review and editing (equal). **Y. M. Chang:** Formal analysis (equal); writing – review and editing (equal). **R. Bond:** Conceptualization (equal); data curation (equal); formal analysis (equal); funding acquisition (equal); investigation (equal); methodology (equal); project administration (equal); resources (equal); supervision (equal); writing – original draft (equal).

### Conflict of interest

None of the authors of this article has a financial or personal relationship with other people or organisations that could inappropriately influence or bias the content of the paper.

## Data Availability

The participants of this study did not give written consent for their data to be shared publicly, so supporting data is not available.
